# Bioremediation of Mercury by* Vibrio fluvialis* Screened from Industrial Effluents

**DOI:** 10.1155/2017/6509648

**Published:** 2017-05-25

**Authors:** Kailasam Saranya, Arumugam Sundaramanickam, Sudhanshu Shekhar, Sankaran Swaminathan, Thangavel Balasubramanian

**Affiliations:** ^1^CAS in Marine Biology, Faculty of Marine Sciences, Annamalai University, Parangipettai 608 502, India; ^2^AVC College of Arts and Science, Mannampandal, Mayiladuthurai 609 305, India

## Abstract

Thirty-one mercury-resistant bacterial strains were isolated from the effluent discharge sites of the SIPCOT industrial area. Among them, only one strain (CASKS5) was selected for further investigation due to its high minimum inhibitory concentration of mercury and low antibiotic susceptibility. In accordance with 16S ribosomal RNA gene sequences, the strain CASKS5 was identified as* Vibrio fluvialis*. The mercury-removal capacity of* V. fluvialis* was analyzed at four different concentrations (100, 150, 200, and 250 *μ*g/ml). Efficient bioremediation was observed at a level of 250 *μ*g/ml with the removal of 60% of mercury ions. The interesting outcome of this study was that the strain* V. fluvialis* had a high bioremediation efficiency but had a low antibiotic resistance. Hence,* V. fluvialis* could be successfully used as a strain for the ecofriendly removal of mercury.

## 1. Introduction

Mercury is one of the most hazardous heavy metals, which is considered as a significant contaminant of the environment. Mercury contamination and its threat to the environment and living organisms is a worldwide problem [1]. It is released into the environment in two ways: natural events and human activities. When compared to the natural processes, human activities have been discharging excessive amounts of mercury into the environment [2]. A primary source of mercury pollution is chloralkali plants, paper pulps, amalgamation industries, fungicides, and paints [3]. The potential of mercury toxicity is only based on its combination with, for example, sulfide, oxide, hydroxide, chloride, and methyl groups. After the incident of Minamata Bay in Japan, mercury poisoning to human health has become evident [4]. A small amount of mercury can have dangerous effects for months in human beings, animals, and plants and even affect the growth of bacteria in microorganisms although some bacteria are capable of surviving and growing in mercury-contaminated sites [5]. Most of the mercury-refinement processes follow common physical and chemical methods, which are highly expensive and have some limitations, whereas biological methods are cost-effective, viable, and friendly to the environment [6]. The use of microorganisms for the removal of metals from contaminated effluents and mining and industrial wastes is considered to be effective because of its efficiency and ecofriendly nature [7]. Recently, the utilization of bacterial biomass under either live or dead conditions for bioremediation has emerged as an efficient, ecofriendly, and cost-effective alternative for the elimination of low concentrations of heavy metals.

The heavy metal and antibiotic-resistant bacteria were found in normal and polluted environments which is a worldwide problem [8]. An array of heavy metals and antibiotics, at concentrations found in different polluted environments, have the potential to coselect both metal-antibiotic-resistant strains and their plasmids [9]. The bacterial agent associated with coselective mechanism of metal-antibiotics is significant at higher threats. Hence, in the present investigation the strain was selected based on the mercury resistance and antibiotic susceptible characteristics. Subsequently, its bioremediation capability was investigated.

## 2. Methods and Materials

### 2.1. Sample Collection

Sediment samples were collected from the common effluent discharge point of the State Industries Promotion Corporation of Tamil Nadu Limited (SIPCOT) industrial area located in the banks of the Uppanar estuary, Tamil Nadu, southeast coast of India. The geographic coordinates of the station are 11°41′45.00′′N latitude and 79°46′05.00′′E longitude. Surface sediments were collected aseptically in triplicate, kept in an insulated box at 4°C, and immediately transferred to the laboratory.

### 2.2. Enrichment and Primary Screening

Sediment samples were added to a 250 ml Erlenmeyer flask containing 100 ml of Zobell Marine Broth (ZMB) at pH 7.1 ± 0.1, incubated for 24 h and centrifuged at 160 rpm at 35°C in a conventional rotary shaker incubator. The bacterial inocula were transferred to a 100 ml ZMB with a supplement of HgCl_2_ and kept in an orbital shaker at 200 rpm for 5 days.

### 2.3. Antibiotic Sensitivity Test

Antibiotic sensitivity test was performed using the disc diffusion method on MH agar with antibiotic disks, by following the methods of CLSI (2013) [10]. Multiple antibiotic profiles of* V. fluvialis* were checked at the following antibiotic concentrations: amoxicillin (10 mcg/disc), bacitracin (10 mcg/disc), erythromycin (15 mcg/disc), amoxicillin (10 mcg/disc), bacitracin (10 mcg/disc), erythromycin (15 mcg/disc), oxytetracycline (30 mcg/disc), novobiocin (30 mcg/disc), cephalothin (30 mcg/disc), vancomycin (30 mcg/disc), and amikacin (10 mcg/disc). Bacterial cultures swabbed on nutrient agar plates and the above-mentioned antibiotics discs were placed in the plates and incubated at 37°C for 24 hrs.

### 2.4. Growth Assessment of CASKS5 with Mercury

The selected* V. fluvialis* overnight culture was inoculated into the nutrient broth, which was supplemented at different concentrations of HgCl_2_ such as 100 *μ*g/ml, 150 *μ*g/ml, 200 *μ*g/ml, and 250 *μ*g/ml in triplicate and kept in a shaking incubator at 37°C for 48 hrs. Growth curves of* V. fluvialis* were observed at periodic time intervals, that is, 2, 4, 8, 16, 24, and 48 hrs using a spectrophotometer (SHIMADZU UV 1800) at 600 nm OD.

### 2.5. Bioremediation Capacity of CASKS5

After the completion of the incubation period, the cultures were centrifuged for 20 mins at 10000 rpm, pellets removed, and supernatants collected and digested with nitric and sulfuric acids. The residual mercury in the medium was analyzed by a cold vapor mercury analyzer (Model MA 5840).

## 3. Results and Discussion

Mercury-resistant bacterial strains were initially screened using the Luria Bertani (LB) medium in the presence of 2.0 *μ*g/ml HgCl_2_ from sediment samples collected at effluent discharge sites.

### 3.1. Isolation, Screening, and Selection of Mercury-Resistant Bacteria

The high mercury-resistant strains were isolated from sediments of industrial discharge sites using several screening techniques. Prolonged exposure to mercury contamination can generate resistance mechanisms in bacteria [11]. During preliminary findings, 31 bacterial isolates exhibited high resistance to mercury. Secondary screening was performed to estimate MIC. The MIC results observed for all the 31 isolates confirmed that the strain CASKS5 had the highest mercury tolerance (100 *μ*g/ml concentration) as shown in Table 1, which is several times greater than that obtained in an earlier study done on the same species by Figueiredo et al. (2016) [12].

### 3.2. Biochemical Characterization and Molecular Identification of CASKS5

The selected strain CASKS5 was morphologically and biochemically characterized as a gram-negative, curved rod-shaped bacterium and tentatively identified as* Vibrio* sp. (Table 2). The 16S rDNA sequence was carried out for CASKS5 and submitted to GenBank (NCBI, 2013) for searching similar published sequences. The accession number of the strain CASKS5 is KM186606.

**Figure 1:**
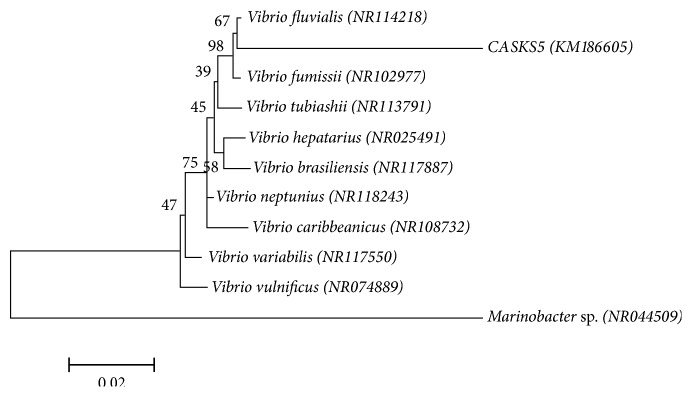
Phylogenetic tree constructed from the 16S rRNA gene sequence of* Vibrio fluvialis* (KM186605) (GenBank accession number KM186605) and closely related organisms using NCBI BLAST. The scale bar represents 0.02 substitutions per nucleotide position.

BLAST analysis revealed that the partial 16S rDNA of CASKS5 had more than 99% similarity to that of* Vibrio fluvialis* strain in NCBI. A phylogenetic tree based on 16S rDNA was constructed using the MEGA 6.0 software to determine the relationship between CASKS5 and* V. fluvialis* (Figure 1). Based on the above characterization, strain CASKS5 was identified as* V*.* fluvialis*.

### 3.3. Antibiotic Profile of* V. fluvialis*

**Figure 2:**
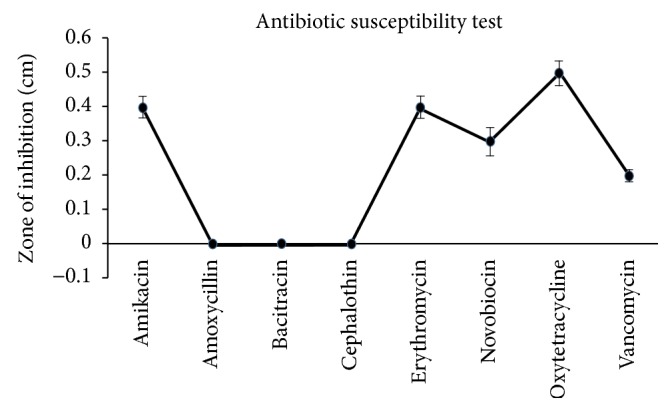
Antimicrobial susceptibility test profile for mercury-resistant bacteria isolate* Vibrio fluvialis* (KM186605).

Earlier investigations dealt with the heavy metal resistance of bacteria from marine environments having high resistance to most of the antibiotics [13, 14]. Nakahara et al. [15] stated that antibiotic- and metal-resistance ability is created by the same plasmid of the bacteria. However, in the present investigation, the results of the antibiotic sensitivity test with eight different antibiotics indicate that the strain CASKS5 was found to be susceptible to the majority of antibiotics, for example, amikacin, erythromycin, novobiocin, oxytetracycline, and vancomycin, although it was resistant to only three antibiotics, namely, amoxicillin, bacitracin, and cephalothin (Figure 2). Much controversy exists within the scientific community over whether metal-resistant bacteria from polluted areas can also have antibiotic resistance. In the present investigation, the selected strain proved to have high resistance to mercury, although with little resistance to antibiotics. The results of Figueiredo et al. (2016) [12] specify that, out of 10 bacterial strains isolated from mercury-contaminated regions of the Tagus estuary, only three have multidrug resistance. They also reported that* V. fluvialis* has resistance only to nalidixic acid among the six antibiotics tested. Hence, the presence of these antibiotic- and mercury-resistant genetic elements in the same gene is again highly contentious.

### 3.4. Effect of Mercury on the Growth of* V. fluvialis*

**Figure 3:**
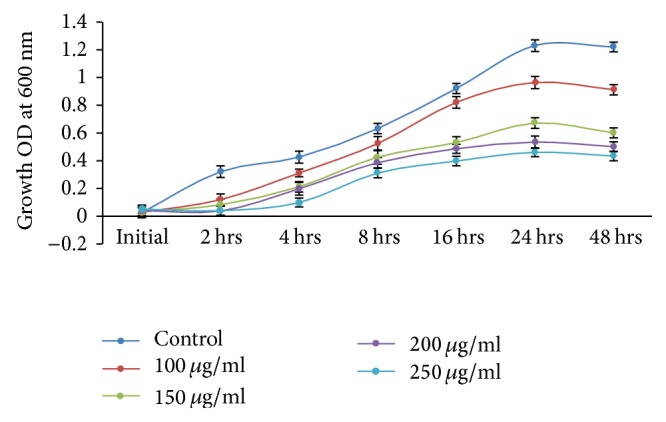
Growth kinetics of* Vibrio fluvialis* (KM186605) in HgCl_2_ (100, 150, 200, and 250 *μ*g/ml) containing medium. Control cultures did not contain any metal ions.

Based on the MIC results, 100 *μ*g/ml was taken as an initial metal concentration for growth curve studies. A significant growth rate of* V. fluvialis* was observed after 24 hrs of incubation in control as well as in culture broth-containing metal, which indicated that gram-negative* V. fluvialis* has developed a potential resistance to mercury. An earlier study by Aram et al. [16] also states that gram-negative bacteria isolated from the Maharloo River, Iran, have higher resistance when compared to gram-positive bacteria. Bioremediation results show that enhanced growth was observed in control as well as solutions with mercury at a lower concentration (100 *μ*g/ml) as compared to higher concentrations (150 *μ*g/ml, 200 *μ*g/ml, and 250 *μ*g/ml), which indicates an increase in the concentration of mercury and a decrease in the growth rate of cells (Figure 3). The present observation corroborates an earlier study described by Zeng et al. (2009) [17] from China.

### 3.5. Mercury-Removal Capacity of* V. fluvialis*

**Figure 4:**
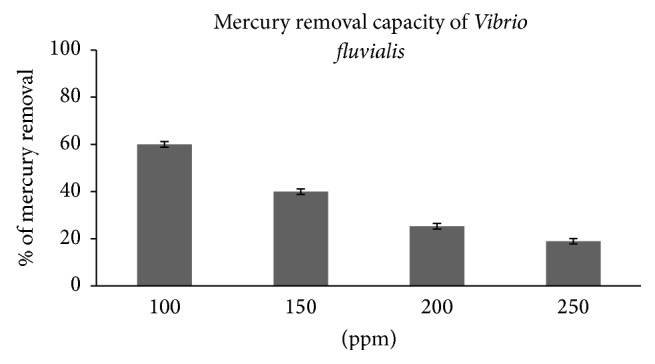
Bioremediation efficiency by* Vibrio fluvialis* (KM186605) with different initial concentration HgCl2 (100, 150, 200, and 250 *μ*g/ml).

Bacteria are a valuable tool to treat mercury because they have vital reactive interfaces for the adsorption of nutrients and foreign contaminants on their cell surface; particularly bacterial membranes act as sites of uptake and exudation and provide plenty of enzymatic actions [18]. In the case of metal contaminants, some bacterial cells uptake metals for their requirements, some of them chelate with metals, and some either reduce or oxidize them [19]. Mercury-remediation capacity of* V. fluvialis* was observed by growth carve at different concentrations of mercury chloride. The highest mercury-remediation rate (60%) was found at a lower mercury concentration of 100 *μ*g/ml after 42 h of incubation. At higher concentrations of 150, 200, and 250 *μ*g/ml, the mercury-removal percentages were 40, 25.33, and 19%, respectively (Figure 4). Some of the previous works of various researchers on mercury bioremediation by bacterial strains are given in Table 3. At a mercury concentration of 10 *μ*g/ml, 89.47% of the mercury was removed by a species under the same genus,* Vibrio parahaemolyticus*, over 40 h of incubation [20]. The results of other bacterial species are as follows:* Bacillus* sp., 68.1% [21];* Bacillus thuringiensis,* 42.7% [22];* Pseudomonas aeruginosa,* 80% [23];* Brevibacterium casei,* 70% [23];* Tetrahymena rostrate,* 40% [24];* Pseudomonas *sp., 65% [25];* Pseudomonas fluorescens,* 34.30% [26] and 42.7% [22];* Pseudomonas aeruginosa,* 80% [23];* Brevibacterium casei,* 70% [23];* Tetrahymena rostrate,* 40% [24];* Pseudomonas *sp., 65% [25];* Pseudomonas fluorescens,* 34.30% [26];* Pseudomonas aeruginosa,* 25% [27];* Klebsiella pneumonia*e, 15% [27]; and the* Enterobacter cloacae* efficiency was below the detectable limit [28]. Some of them observed a better removal of mercury than in the present study. However, the concentration level used in other studies was lower than that in our present investigation which is shown in Table 3.

## 4. Conclusion

The results demonstrate that* V. fluvialis* has strong ability to detoxify mercury from mobile solutions. In addition, mercury and antibiotic resistance were appraised in detail for the selected strain. Generally, metal-resistant strains exhibit a strong antibiotic resistance; nevertheless, the present findings specify that the isolate CASKS5 has resistance to only a few antibiotics. Hence, the strain CASKS5 can be utilized as a good chelating agent for the removal of mercury from contaminated effluents because of its high efficiency. Further studies have to be performed to find out the mechanism behind the removal of mercury by* V. fluvialis* and also to examine the preference for heavy metal uptake by this bacterium in the presence of other heavy metals.

## Figures and Tables

**Table 1:** Similarity of minimum inhibitory mercury concentration value against that observed in the present strain *Vibrio fluvialis* to those reported elsewhere.

Strain	MIC (*μ*g/ml)	Location	Reference
*Vibrio fluvialis*	1	Tagus Estuary (Portugal)	Figueiredo et al., 2016
*Vibrio natriegens*	20	Coastal sediments, Bushehr, Iran	Jafari and Cheraghi, 2014
*Vibrio* sp.	12–16	Chesapeake Bay	Walker and Colwell, 1974
*Vibrio* sp.	2.71	Mai Po Nature Reserve, Hong Kong	Zhang et al., 2006
*Vibrio parahaemolyticus *	45	Coastal sediments, Bushehr, Iran	Jafari and Cheraghi, 2014
*Vibrio fluvialis*	100	Parangipettai coast (India)	Present study

**Table 2:** Biochemical characteristics of strain mercury resistant bacterial strain CASKS5.

Tests	Results
Morphology	
Gram reaction	−ve
Shape	Rod

Biochemical reactions	
Citrate utilisation	+
Indole	+
Methyl red	−
Nitrate reduction	+
Oxidase	+
Catalase	+
Voges Proskauer	−
Gelatin	+

Carbohydrate utilisation	
Glucose	+
Arabinose	−
Sucrose	+

**Table 3:** Bioremediation efficiency of mercury by *Vibrio fluvialis* (CASKS5) compared with different bacterial species isolated from elsewhere.

Location	Species	Concentration of mercury used for the experiment, *μ*g/ml	Bioremediation efficiency (in percentage)	References
Bushehr (Iran) coast	*Vibrio parahaemolyticus*	10	>89.47	Jafari et al., 2015
Urías estuary, Sinaloa, Mexico	*Bacillus *sp.	10	68.1	Green-Ruiz, 2006
	*Bacillus thuringiensis*		42.7	Hassen et al., 1998
Sialkot (Pakistan) pond	*Brevibacterium casei*	50	70	Rehman et al., 2007
Sialkot (Pakistan) pond	*Pseudomonas aeruginosa*	50	80	Rehman et al., 2007
Kasur (Pakistan) ponds	*Tetrahymena rostrata*	100	40	Muneer et al., 2013
Sialkot (Pakistan) ponds	*Pseudomonas *sp.	100	65	Rehman et al., 2008
South Korea municipal waste water treatment plant	*Pseudomonas fluorescens*	30	34.3	Noghabi et al., 2007
King Abdul-Aziz Medical City, Jeddah, Saudi Arabia,	*Pseudomonas aeruginosa*	150	25	Al-Garni et al., 2010
King Abdul-Aziz Medical City, Jeddah, Saudi Arabia,	*Klebsiella pneumonia*e	100	15	Al-Garni et al., 2010
West Coast of India	*Enterobacter cloacae*	100	0	Iyer et al., 2005
Cuddalore coast (India)	*Vibrio fluvialis*	100	60	Present study
